# PAN-811 prevents chemotherapy-induced cognitive impairment and preserves neurogenesis in the hippocampus of adult rats

**DOI:** 10.1371/journal.pone.0191866

**Published:** 2018-01-25

**Authors:** Zhi-Gang Jiang, Gordon Winocur, J. Martin Wojtowicz, Olga Shevtsova, Steven Fuller, Hossein A. Ghanbari

**Affiliations:** 1 Panacea Pharmaceuticals, Inc., Gaithersburg, Maryland, United States of America; 2 Department of Psychology, Trent University, Peterborough, Ontario, Canada; 3 Rotman Research Institute, Baycrest Centre, Toronto, Ontario, Canada; 4 Departments of Psychology and Psychiatry, University of Toronto, Toronto, Ontario, Canada; 5 Department of Physiology, University of Toronto, Toronto, Ontario, Canada; Technion Israel Institute of Technology, ISRAEL

## Abstract

Chemotherapy-induced cognitive impairment (CICI) occurs in a substantial proportion of treated cancer patients, with no drug currently available for its therapy. This study investigated whether PAN-811, a ribonucleotide reductase inhibitor, can reduce cognitive impairment and related suppression of neurogenesis following chemotherapy in an animal model. Young adult rats in Chemo and Chemo+PAN-811 groups received 3 intraperitoneal (*i*.*p*.) injections of methotrexate (MTX) and 5-fluorouracil (5-FU), and those in Saline and Saline+PAN-811 groups received equal volumes of physiological saline at 10-day intervals. PAN-811 in saline was delivered through *i*.*p*. injection, 10 min following each saline (Saline+PAN-811 group) or MTX/5-FU (Chemo+PAN-811 group) treatment, while equal volumes of saline were delivered to Saline and Chemo groups. Over Days 31–66, rats were administered tests of spatial memory, nonmatching-to-sample rule learning, and discrimination learning, which are sensitive to dysfunction in hippocampus, frontal lobe and striatum, respectively. On Day 97, neurogenesis was immnunohistochemically evaluated by counting doublecortin-positive (DCX^+^) cells in the dentate gyrus (DG). The results demonstrated that the Chemo group was impaired on the three cognitive tasks, but co-administration of PAN-811 significantly reduced all MTX/5-FU-induced cognitive impairments. In addition, MTX/5-FU reduced DCX^+^ cells to 67% of that in Saline control rats, an effect that was completely blocked by PAN-811 co-administration. Overall, we present the first evidence that PAN-811 protects cognitive functions and preserves neurogenesis from deleterious effects of MTX/5-FU. The current findings provide a basis for rapid clinical translation to determine the effect of PAN-811 on CICI in human.

## Introduction

Though target-oriented immunotherapy is an important direction in the treatment of cancer, small chemical molecules are still widely used as anticancer drugs in clinical cancer therapy. Such drugs are usually associated with severe side effects that can affect quality of life and pose difficulties for the continuation of therapy. One of the major adverse effects is chemotherapy-induced cognitive impairment (CICI, also known as ‘chemobrain’ or ‘chemo fog’), that occurs during and after chemotherapy in up to 75% of cancer survivors treated with anti-cancer drugs [1, 2]. CICI impairs multiple cognitive functions, including attention, reasoning, learning, memory, problem solving, as well as visuospatial skills [3–5]. Clinically, CICI can last for up to 10 years, post-treatment [6, 7]. Despite the seriousness of the problem, there are no therapeutic drugs currently available for treating CICI [8]. To improve the quality of life for patients and to ensure continuation of cancer therapy, the identification and development of a drug for treatment of CICI is imperative.

The anticancer drugs methotrexate (MTX) and 5-fluorouracil (5-FU) are antimetabolites that function in suppression of cell mitosis and proliferation, and thus, are commonly used in clinical treatment of various cancers, such as breast cancer. Both MTX and 5-FU can pass the blood-brain barrier (BBB) in amounts that are sufficient to cause cognitive impairment [2, 9–11], possibly by increasing oxidative stress (OS) [10, 12, 13]. In line with this mechanism, we previously demonstrated that MTX/5-FU-induced *in vitro* neurotoxicity is OS-dependent [14]. Increased OS, which is associated with various forms of chemotherapy [10], can elicit apoptosis of primary neural precursor cells and reduce the number of proliferation cells in the hippocampus of rodents [15, 16]. Previous reports have related hippocampus-mediated memory loss following treatment with MTX and 5-FU to the drugs’ effects on neurogenesis levels [17–20].

There has been very little research into remediation strategies for relieving CICI. Although some encouraging results have been reported in preclinical studies involving the anti-depressant fluoxetine, a selective serotonin reuptake inhibitor [21] and the cognitive enhancing drug donepezil, an acetylcholinesterase inhibitor [22], both primarily moderate the function loss. In contrast, PAN-811 is a demonstrated neuroprotectant that blocks neurotoxic pathways, and here, we investigate its therapeutic effects on CICI in a rat model. PAN-811, chemical name 3-aminopyridine-2-carboxaldehyde thiosemicarbazone (3-AP, also called Triapine), is a ribonucleotide reductase inhibitor, originally designed for cancer therapy [23–25] and tested in Phase 1 and 2 clinical trials with a favorable safety profile [26–30]. PAN-811 has shown a capability of scavenging stable free radicals in a cell-free environment, and the ability to inhibit H_2_O_2_-induced neurotoxicity [31–33]. We have recently demonstrated that PAN-811 blocks OS-dependent *in vitro* neurotoxicity of 5-FU or MTX [14].

Cognitive performance following MTX and 5-FU treatment has been evaluated in a well-established rodent model using tests that are sensitive to dysfunction in different brain regions. Deficits have been reported on hippocampus-dependent tests of spatial memory, non-matching-to-sample rule-learning that is controlled by the frontal lobes, and discrimination learning that is associated with the corpus striatum [17, 18, 34–38]. We hypothesized that PAN-811 would protect animals treated with MTX/5-FU from cognitive impairment, as measured by these tests. As well, since suppression of neurogenesis is thought to be a mechanism underlying MTX/5-FU-induced impairment of cognitive processes mediated by the hippocampus, we examined new cell production in the dentate gyrus to determine if PAN-811 is capable of counteracting this suppressive effect. The results show that PAN-811 prevents cognitive deficits resulting from chemotherapy with MTX/5-FU in our model and preserves neurogenesis in the dentate gyrus.

## Materials and methods

### Materials

PAN-811∙Cl∙H_2_O was produced by Kimia Corp, Santa Clara for Panacea Pharmaceuticals Inc. MTX and 5-FU were purchased from Wyeth Canada, Thornhill, Ontario, and Mayne Pharma, Kirkland, Quebec, respectively.

### Animal model and treatment

The study was conducted using healthy female adult Long Evan rats (Charles River Laboratories, St. Constant, Quebec), 3 months old at the beginning of the experiment. After one week in quarantine, they were transferred to standard laboratory cages with food and water always available. All rats were maintained on a 12-hour light-dark schedule, with lights on between 8:00PM and 8:00AM.

The experimental protocol and all handling procedures were approved by the Trent University and University of Toronto Animal Care Committees, and conformed to requirements of the Canadian Council on Animal Care. The rats were examined regularly by a veterinarian throughout the experiment.

**Fig 1:**
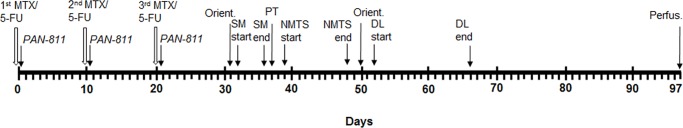
Experimental timeline. A combination of 5-FU and MTX or equal volume of physiological saline was administered to 3-month old rats, *i*.*p*., 3 times at 10-day intervals, and PAN-811 or equal volume of saline was injected, *i*.*p*., 10 min following each anticancer drug administration. Chemo: MTX/5-FU; PAN: PAN-811; Inject: injection; Orient.: Orientation; SM: spatial memory test; PT: probe test; NMTS: nonmatching-to-sample test; DL: discrimination learning test; Perfus.: perfusion; IHC: immunohistochemistry.

Initially, 46 rats were assigned randomly to 4 groups—saline plus saline (Saline, N = 10), chemotherapy plus saline (Chemo, N = 13), saline plus PAN-811 (Saline+PAN, N = 10), and chemotherapy plus PAN-811 (Chemo+PAN, N = 13). Rats receiving chemotherapy in Chemo or Chemo+PAN groups were administered 37.5mg/kg MTX and 50mg/kg 5-FU dissolved in physiological (normal) saline, *i*.*p*., 3 times with 10-day intervals. Control rats in Saline and Saline+PAN groups were injected with equal volumes of saline at the same time points. For treatment, in Chemo+PAN and Saline+PAN groups, rats were administered 12mg/kg PAN-811∙Cl∙H_2_O (a salt format of PAN-811) dissolved in normal saline, *i*.*p*., also 3 times, but 10 min following each administration of the anticancer drugs or saline. Likewise, rats in Chemo and Saline groups were administered equal volumes of the normal saline, *i*.*p*., 3 times, 10 min following each administration of anticancer drugs or saline (Table 1). Fig 1 provides the overall study timeline.

**Table 1:** Regime at each treatment day (3 sets of injections at 10-day intervals).

Groups	Insult	Treatment (10 min later)
***Chemo***	MTX/5-FU	Saline
***Chemo+PAN***	MTX/5-FU	PAN-811
***Saline+PAN***	Saline	PAN-811
***Saline***	Saline	Saline

The doses for all drugs were selected on the basis of dose-response tests for tolerance and toxicity. The dosages were well tolerated and did not influence appetite or activity levels. The only noticeable effect was a small amount of hair loss in a few rats that had received MTX/5-FU.

### Cognitive tests

The study utilized the following cognitive tests to investigate various aspects of learning and memory.

#### Spatial learning and memory (SM)

The SM test is a widely used, highly sensitive test of hippocampal dysfunction [38]. The test was conducted in a circular pool (130 cm diameter and 30 cm high), located in the center of a standard testing room. The pool was filled with opaque water and maintained at 21^o^ C. An inverted flowerpot (10cm diameter), situated a few cm below the surface, served as a platform on which the rats could climb to escape the water. Throughout testing, the water was cleaned after each trial and changed every 2–3 days. The pool was divided into 6 zones of approximately equal size. Swimming patterns were monitored by an overhead video camera connected to a recorder and data processing system. The system recorded swimming routes that were used to count errors. An error was recorded each time the rat entered a zone not containing the platform. On Day 31, the rats received one day of orientation training (5 trials/day) in which they learned to swim to the platform that was visible and in a different location on each trial. SM testing began the following day (Day 32). The platform was now submerged and always located in the center of the northeast zone. At the start of each trial, the rat was placed in the water at the edge of the pool, facing the wall, at a different location, but never in the northeast zone. A trial continued until the rat mounted the platform with all four paws. If it failed to find the platform in 60 sec., it was guided to the platform, and assigned an error score of 15. After 20 sec. on the platform, the rat was placed under a heat lamp in a holding cage to await the next trial. Each rat received 5 such trials/day for 5 consecutive days (Days 32–36). One rat assigned to the Chemo+PAN group died following of MTX/FU injection, and one rat was removed from Saline group due to a handling error. Therefore, 44 rats completed SM test and probe test (PT. Chemo: n = 13; Chemo+PAN: n = 12; Saline+PAN: n = 10; Saline: n = 9).

PT or probe trial provided an additional test of memory for the location of the platform. On Day 37, trials 1 & 2 were conducted in the usual manner. On the third trial, the platform was removed and the rats were allowed to swim for 60 sec. Time spent in the target zone for each group was expressed as a percentage of the 60-sec period. The number of rats in each group was same as that in SM test. Data were expressed as arithmetic means ± SEM.

#### Nonmatching-to-sample learning (NMTS)

The NMTS test consists of a series of paired sample (or study)—test trials in a water maze. The stimuli for the sample and test trials were black and white cylinders (30 cm long x 3 cm in diameter), suspended 5 centimeters above the surface of the water. In the sample trials, one of the stimuli signaled the platform’s location. In the subsequent test trial, the sample stimulus was presented along with the other stimulus in new locations. In the test trial, the cylinder that was not present during the preceding sample trial now signaled the platform’s location. NMTS and related rule-learning tasks incorporate conditional and working memory components and are known to be sensitive to frontal-lobe impairment [35].

NMTS testing began on Day 39. For the sample trials, the rat was placed in the southeast zone of the pool and allowed to swim to the submerged platform under a sample cylinder. The rat remained on the platform for 20 sec. and then placed under the heat lamp while the platform together with the cylinders were re-located to different zones. For the test trial, which began 10 sec. later, the rat was placed in the pool at a different location (with the exceptions of the zone containing either cylinder and the target zone in the preceding sample trial), and allowed to swim to the submerged platform. If the rat failed to find the platform within 60 sec., it was guided to the platform and given an error score of 15. After 20 sec. on the platform the rat was placed under the heat lamp, to await the next pair of trials. Ten sessions, each consisting of 5 pairs of sample and test trials, were administered each day over 10 days (Days 39–48). One rat assigned to the Chemo+PAN group died, 17 days following final drug injection and, as a result, did not participate in NMTS and DL testing. Therefore, 43 rats completed NMTS test (Chemo: n = 13; Chemo+PAN: n = 11; Saline+PAN: n = 10; Saline: n = 9). Data from test trials (not sample trials) were used for result judgement.

#### Discrimination learning (DL)

The DL test requires the rat to discriminate between horizontal vs vertical, black and white striped cylinders (30 cm long x 3 cm in diameter) in order to find the submerged platform. The task measures non-conditional, stimulus-response learning and is sensitive to impairment in the striatal system [34].

In this test, the pool was fitted with a gray, plastic cross-maze with walls that extended 10 cm above the surface of the water. Each arm of the maze was 55 cm long. Orientation training started on Day 50 (Day 30 following the final drug administration), and lasted for 2 days. For each orientation trial, the rat was placed in the pool at the end of one of the arms and allowed to swim to a submerged platform which was located at the end of each goal arm. Each orientation session consisted of 5 trials/day. For these trials, there was no discrete cue to direct the animal.

DL began the day following orientation (on Day 52 or Day 32 following the final drug administration), and consisted of 10 trials/day. On each trial, the rat was placed in the pool at the end of one of the arms and allowed to swim to the choice point, where it encountered the black and white cylinders. For half the rats, the cylinder with horizontal stripes was positive, and for the other half, the cylinder with vertical stripes was positive. The selection of the start arm for each trial and the positioning of the cylinders were determined by a random schedule. A submerged platform was located at the end of the correct arm. The rat made a correct response if, at the choice point, it turned in the direction of the correct stimulus and swam down that arm. An error was scored when a rat entered an incorrect arm with its whole body (less the tail) or left the correct arm. Between trials of orientation training and discrimination learning, animals were placed under the heat lamp to await the next trial.

Rats were tested on the DL task until they reached a criterion of 0.5 errors/trial/day averaged over two consecutive days. Testing was terminated if an animal failed to reach this criterion after 15 days (by the end of Day 66 or the end of Day 46 following final drug administration), and a score of 150 was assigned. The number of rats in each group was same as that in NMTS test.

### Immunohistochemistry

In order to examine and compare the level of adult neurogenesis in the 4 treatment groups, the brain sections were stained by an immunohistochemistry method using Doublecortin (DCX) antibody. Doublecortin is a reliable marker of immature neurons and has become a standard method for quantification of neurogenesis. Rats were sacrificed on Day 97 by an overdose *i*.*p*. injection of Euthansol (Day 77 following the final drug administration). Brain tissue was fixed by pre-intracardiac perfusion and post-fixed with 4% paraformaldehyde for 24 hrs. Brains were cut in half and the hippocampus was isolated from the right hemisphere of each rat. Isolated hippocampi were sectioned serially along the dorso-ventral axis using a vibratome (VT1000S, Leica Microsystems, Heidelberg, Germany) into sections 30 μm thick. The sections were stored in phosphate-buffered saline (PBS) with 0.1% sodium azide for further processing. Twelve sections from each animal were sampled using a systematic random sampling procedure previously described [38]. DCX staining was performed on free-floating sections. Importantly, sections were rinsed extensively in PBS before processing and between each incubation. All primary and secondary antibody incubations were conducted in PBS containing 0.3% Triton X-100. The sections were incubated with a primary goat anti-DCX antibody (1:200, sc-8066, Santa Cruz Biotechnology, 24 hours at 4°C), followed by secondary antibody donkey anti-goat IgG Alexa 488 (1:200; A11055, Life Technologies; 2 hours at room temperature (RT) in the dark). Then sections were mounted onto glass slides using double-distilled water (ddH_2_O), and coverslipped using PermaFluo (Thermo Scientific, Freemont, CA, USA). Immunohistochemical controls included the omission of primary antibody, which resulted in lack of staining at the corresponding wavelength. Sections were examined and immunolabelled cells in the dentate gyrus (DG) of hippocampus were counted using a Leica TCS-SL confocal microscope (Leica Microsystems (Canada) Inc.; Richmond Hill, ON, Canada) with a 63× oil immersion objective lens. The experimenter was blinded with respect to the group and animal identification number to avoid bias. Immuno-positive cells (DCX^+^) were counted in the subgranular zone (SGZ) of DG. The SGZ was defined as a two-cell width wide (approximately 20 μm) region just below the granule cell layer (GCL). All cells within each section were counted, but excluding top and bottom surfaces of the sections in order to avoid counting cells that were dissected or damaged. The average number of cells per section was multiplied by the total number of sections to obtain total cell numbers per DG [38, 39].

### Data expression and statistical analysis

The data from rats in each group were expressed as geometric mean ± standard error (SEM) for the SM test, based on a log distribution profile of the data, and as arithmetic means ± SEM for all other tests. The data in all tests were verified normal distributions and results were statistically evaluated at a significance level of 1% with One-Way ANOVA followed by the Tukey HSD test for each day of a time-course test and also with T-Test by using software VASSARSTATS (http://vassarstats.net/index.html). Figure symbols are as follows: #, *p* < 0.05; ##, *p* < 0.01; ###, *p* < 0.005 for comparisons with the Saline group; *, *p* < 0.05; **, *p* < 0.01; ***, *p* < 0.005 for comparisons with the Chemo group.

## Results

### PAN-811 significantly reduces MTX/5-FU-induced impairment in the spatial memory (SM) test

**Fig 2:**
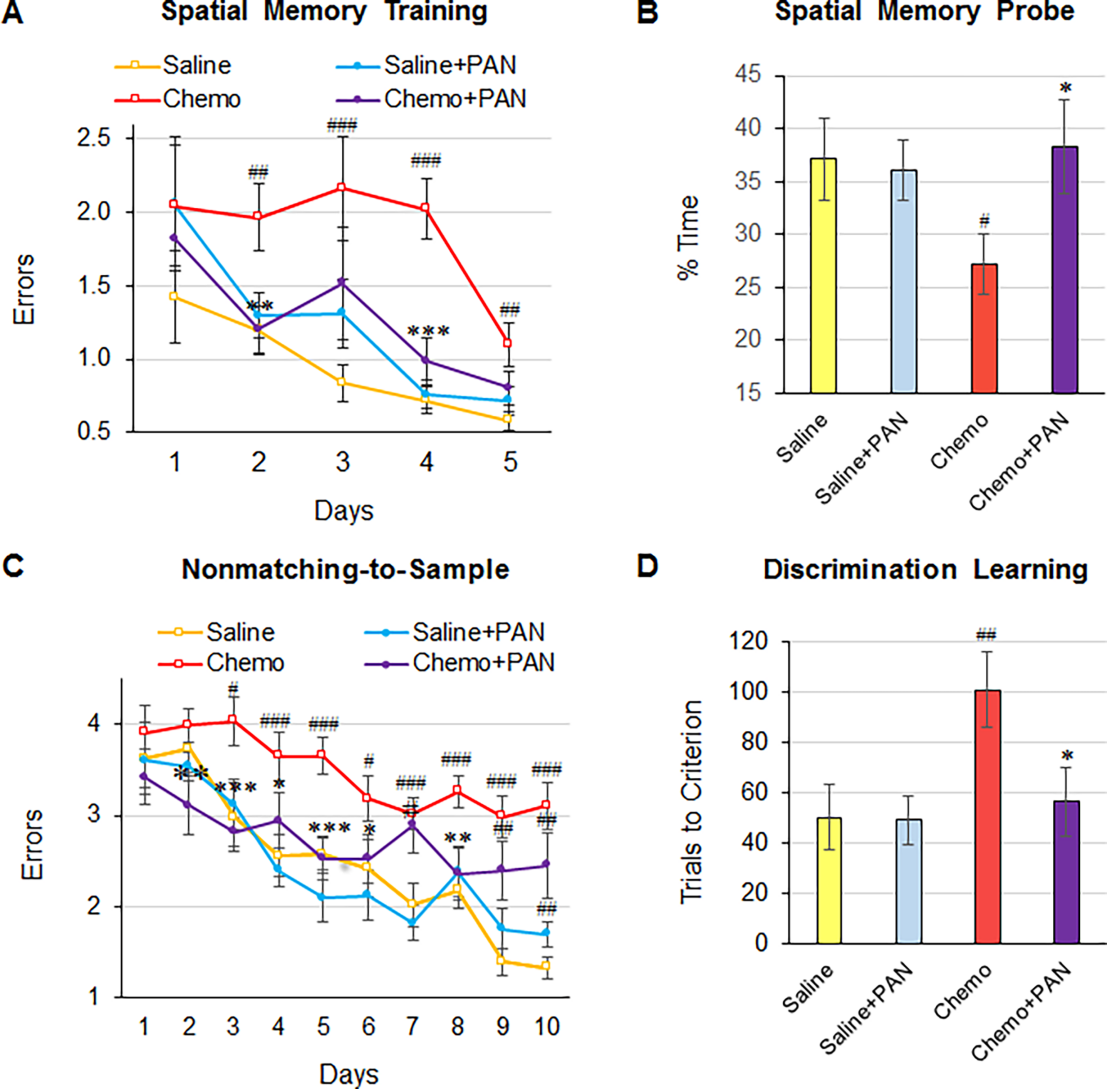
PAN-811 reverses MTX/5-FU-induced cognitive deficits. Rats received 3 *i*.*p*. injections of MTX/5-FU or equal volume of saline at 10-day intervals, followed with PAN-811 or saline *i*.*p*. delivery. (A) Effects of MTX/5-FU (Chemo) and PAN-811 (Saline+PAN and Chemo+PAN) on spatial memory training. Data are presented as geometric means ± SEM. (B) Effects of MTX/5-FU and PAN-811 on performance in spatial memory probe test. Data are expressed as arithmetic mean ± SEM. (C) Effects of MTX/5-FU and PAN-811 on performance in nonmatching-to-sample test. Data are expressed as arithmetic mean ± SEM. (D) Effects of MTX/5-FU and PAN-811 on discrimination learning. Data from 4 groups per day were statistically analyzed with One-Way ANOVA followed by Tukey HSD test, and paired group also analyzed with T-Test. Figure symbols are #, *p* < 0.05, ##, *p* < 0.01 and ###, *p* < 0.005 compared with Saline control; * *p* < 0.05, ** *p* < 0.01 and *** *p* < 0.005 compared with Chemo group.

Spatial learning and memory are closely related to hippocampus function. There were no differences between groups in terms of latency to reach and climb on the visible platform during orientation training (Table 2; *F*_3, 41_ = 0.47, *p* = 0.71). Similarly, on the first day following orientation, no statistically significant difference existed between the 4 groups (Fig 2A. *T*_21_ = -0.63, *p* > 0.05 by T-Test; *F*_3, 41_ = 0.23, *p* > 0.05 by ANOVA). However, on Days 2–4 of the SM test, the Chemo group made more errors than the Saline or other groups in finding the platform on Day 2 (*T*_21_ = -1.95, *p* < 0.05 by T-Test; *F*_3, 41_ = 3.52, *p* < 0.05 by ANOVA), Day 3 (*T*_21_ = -3.23, *p* < 0.005 by T-Test; *F*_3, 41_ = 3.27, *p* < 0.05 by ANOVA) and Day 4 (*T*_21_ = -5.46, *p* < 0.005 by T-Test; *F*_3, 41_ = 16.93, *p* < 0.005 by ANOVA). The Chemo group improved dramatically on Day 5, but still showed a statistically significant difference from the Saline or other groups (*T*_21_ = -2.9, *p* < 0.005 by T-Test; *F*_3, 41_ = 3.63, *p* < 0.05 by ANOVA). These data demonstrate that chemotherapy impaired performance during the spatial memory learning stage. By contrast, the Saline+PAN group did not differ from the Saline control group (no statistically significant difference between these two groups) on most test days, except that the error rate in the Saline+PAN group was slightly higher than that of Saline group on Day 3 (*T*_18_ = -1.89, *p* < 0.05 by T-Test, but not by Tukey HSD Test following One-Way ANOVA). Interestingly, in the Chemo+PAN group, the errors in finding the platform were significantly lower than those in the Chemo group on Day 2 (*T*_23_ = 2.72, *p* < 0.01 by T-Test; *F*_3, 41_ = 3.52, *p* < 0.05 by ANOVA) and Day 4 (*T*_23_ = 3.99, *p* < 0.005 by T-Test; *F*_3, 41_ = 16.93, *p* < 0.01 Tukey HSD Test following ANOVA). Although there was no statistically significant difference between these two groups on Day 3 and Day 5, the mean number of errors made by the Chemo+PAN group was much lower than that in the Chemo group and not significantly different from the Saline group. These indicated a beneficial effect of PAN-811 on chemotherapy-treated rats.

**Table 2:** Mean latencies (sec.) for all groups to find visible platform over 5 trials of orientation training.

Groups	*Chemo*	*Chemo+PAN*	*Saline*	*Saline+PAN*
***Mean***	6.70	7.16	7.38	7.07
***SEM***	0.28	0.50	0.45	0.45

The PT, which was carried out on Day 6 of the SM test, measured time spent in the zone where the platform was previously present, provides an additional measure of hippocampus-sensitive spatial memory (Fig 2B). As can be seen in Fig 2B, the Chemo group spent significantly less time in the target zone than the Saline group (*T*_21_ = 2.5, *p* < 0.05 by T-Test). As in the SM test, PAN-811 treatment protected rats against the effects of chemotherapy on spatial memory. The % time spent by the Chemo+PAN group in the target zone was significantly higher than that in Chemo group (*T*_22_ = -2.86, *p* < 0.005 by T-Test), and did not differ from the Saline group (*T*_19_ = 0.7, *p* > 0.05 by T-Test). Thus PAN-811 efficiently protected SM function in against MTX/5-FU insult.

### PAN-811 reduces MTX/5-FU-induced deficits in NMTS rule learning

The NMTS test was used to measure conditional learning and working memory, which reflects frontal lobe function. From Days 3–10 of NMTS testing, the Chemo group demonstrated significantly higher errors than the Saline group (Fig 2C). PAN-811 treatment did not affect performance in comparison with the Saline control (no statistically significant difference between the two groups) on test Days 1–9, except for a slight increase in errors on Day 10 (*T*_18_ = -2.65, *p* < 0.01 by T-Test but not by One-Way ANOVA). Co-administration of PAN-811 significantly reduced MTX/5-FU-induced errors to the levels of those in the Saline group on Days 2–6 and Day 8. The numbers of errors in the Chemo+PAN group were lower than those in the Chemo group on Days 7, 9 and 10, but were not statistically significant. Generally, then, PAN-811 robustly suppressed MTX/5-FU- induced errors in the NMTS test.

### PAN-811 reduces MTX/5-FU-induced deficit in discrimination learning

The DL test assessed discrimination capability that is sensitive to impairment of the striatal system. The measure of learning was the number of trials required to achieve a criterion of 0.5 errors/trial on two consecutive days of testing. The number of trials to criterion in the Chemo group was significantly greater than that in the Saline group (Fig 2D; *T*_20_ = -2.733, *p* < 0.01 by T-Test; *F*_3, 39_ = 4.65, *p* < 0.05 by Tukey HSD Test following One-Way ANOVA and). PAN-811 by itself did not affect discrimination learning in comparison with Saline control (no statistically significant difference between the two groups). PAN-811 significantly reduced the number of the trials to criterion in the Chemo+PAN group (*T*_22_ = 2.49, *p* < 0.05 by T-Test; *F*_3, 39_ = 4.65, *p* < 0.05 by Tukey HSD Test following ANOVA, compared with Chemo group) down to the level in the Saline group (no statistically significant difference between Saline and Chemo+PAN).

### PAN-811 blocks MTX/5-FU-elicited damage to neurogenesis in the subgranular zone

**Fig 3:**
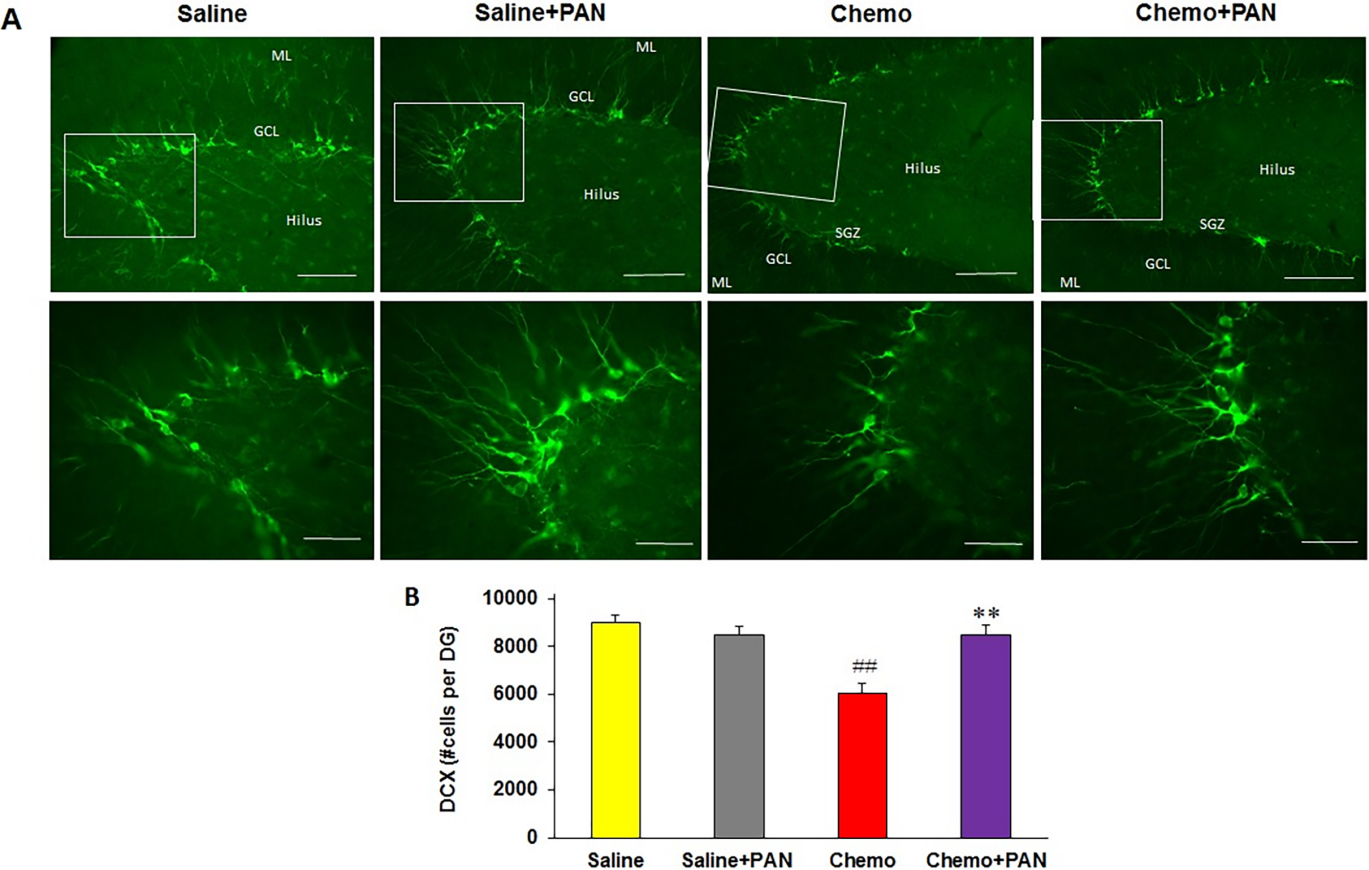
PAN-811 preserves neurogenesis against MTX/5-FU insult. DCX staining of hippocampus on Day 97. (A) Representative fluorescent image of DCX, in which the abbreviations are: SGZ, subgranular zone; GCL, granule cell layer; and ML, molecular layer. Scale bars for photos in the top row, 100 μm; Scale bars for photos in the bottom row, 50 μm. (B) Quantification of DCX^+^ cells in SGZ. Chemo+PAN: Chemo+PAN-811. Data were analyzed with both One-Way ANOVA as well as T-Test. Figure symbols are ##, *P* < 0.01 compared with Saline control; **, *P* < 0.01 compared with Chemo group.

DCX is a protein that expresses in both neural precursor cells and immature neurons, which involves neurogenesis in adult brain. DCX positive cells were labeled with green fluorescence (Fig 3A), which not only shown in cell body but also in cell processes in the subgranular zone (SGZ) of DG. The density of DCX^+^ cells in the Saline+PAN group was about same as that in the Saline group. However, the number of DCX^+^ cells was clearly less in the Chemo group, in comparison with that in the Saline control. There were more DCX^+^ cells in the Chemo+PAN group than in the Chemo group. The density of cell processes in the PAN and Chemo+PAN groups appeared also higher. DCX^+^ cells in SGZ were manually quantified in blind way to avoid bias (Fig 3B). MTX/5-FU in Chemo group reduced number of DCX^+^ cells to 67% of that in the Saline group (*F*_3, 16_ = 13.27, *p* < 0.01 by Tukey HSD Test following ANOVA). PAN-811 did not cause any decrease of DCX^+^ cells in number. In the Chemo+PAN group, the number of DCX^+^ cells was significantly higher than that in the Chemo group (*F*_3, 16_ = 13.27, *p* < 0.01 by Tukey HSD Test following ANOVA) and 94% of that in the Saline control (no statistically significant difference between two groups). Thus, PAN-811 provided a full preservation of neurogenesis in the SGZ under MTX/5-FU stress.

## Discussion

In line with previous findings in the rat [17, 40] and the mouse [36, 37], deficits were observed in spatial learning and memory, NMTS rule learning, and discrimination learning following *i*.*p*. delivery of MTX/5-FU. The impairment was widespread, affecting a range of cognitive processes associated with the hippocampus [38], frontal lobes [35], and corpus striatum [34], respectively. MTX/5-FU-elicited CNS impairment could also be enduring. The measurement of cognitive functions started from Day 31 and finished by the end of Day 66 (the end of the final test), and cognitive dysfunction manifested through whole time period. Additionally, histopathology was examined on Day 97 and MTX/5-FU-induced cellular changes were clearly observed at that time.

The combination of MTX and 5-FU has dual effects. It can damage both cancer cells and neural stem cells [17, 18]. The former introduces a therapeutic benefit with respect to the disease while the latter has negative side effects on the nervous system. Second, OS is a common factor in cytotoxicity, and both MTX and 5-FU increase *in vivo* OS and result in damage [41–45]. To gain insight into underlying mechanisms for MTX/5-FU-elicited cognitive impairment, we examined the effect of chemotherapy on neurogenesis in adult rat brains by labeling neural precursor cells and immature neurons with DCX. In the granular zone of the DG of hippocampus, MTX/5-FU significantly reduced the number of DCX^+^ cells, indicating a deleterious effect on the neurogenesis of adult brain. The present results, along with those of previous studies [19, 20, 40], indicate that suppression of neurogenesis in the dentate gyrus is an underlying mechanism of chemotherapy-induced impairment on the SM task, a widely accepted test of hippocampal function. The mechanisms underlying the frontal lobe- and striatum- related impairment are less clear. Reactive oxygen species (ROS) can exert its neurotoxic effect via induction of neuroinflammation. A recent study provided evidence that alteration in brain volume and dysregulation of neuroinflammatory cytokine activity are associated with such deficits [46]. The anatomical connection between the frontal lobes and corpus striatum suggest that the Chemo group’s poor performance on the DL task may be associated with similar mechanisms although, in the absence of firm evidence, this hypothesis must be considered speculative.

PAN-811 at a dose of 12mg/kg does not introduce any neurotoxicity by comparison with control group, as shown with cognitive tests and IHC examination. Furthermore, PAN-811 at this dose does not show fatal toxic to rats, since rat number in Saline+PAN group remained same as that in Saline group by the end of experiment.

Functionally, systemic delivery of PAN-811 via the *i*.*p*. route achieved a significant preservation of hippocampus-controlled cognitive function under MTX/5-FU stress, as demonstrated by suppression of MTX/5-FU-elicited error increase in the SM test and by blocking MTX/5-FU-induced percent time reduction in target zone during the PT test of spatial memory. Similarly, PAN-811 also provided effective neuroprotection from MTX/5-FU stress on frontal lobe and striatal function, as reflected in improved performance in the Chemo+PAN group on the NMTS and DL tests. This is the first demonstration that PAN-811 can protect against MTX/5-FU-induced cognitive impairment in an animal model.

In related study, the anticancer drug cyclophosphamide treatment induced significant performance-based decrements on behavior tasks whereas intrahippocampal transplantation of human neural stem cells resolved all cognitive impairment in rats [47]. In the present study, PAN-811 fully preserved neurogenesis in the DG of hippocampus under MTX/5-FU insult. Since MTX/5-FU most likely damaged neurons by oxidative stress in the same way as it is toxic to cultured neurons [14], the preservation of these neurons by PAN-811 may be via suppression of MTX/5-FU-elicited oxidative stress. In support of this hypothesis, our previous study revealed that PAN-811 can inhibit ischemic or hypoxic neurotoxicity to post-mitotic neurons by scavenging free radicals and suppressing OS [31]. PAN-811 has been shown to protect neurons from H_2_O_2_-induced cell death [32, 33] as well.

## Conclusions

In conclusion, this study demonstrates that PAN-811 efficiently reduced MTX/5-FU-elicited cognitive impairment and preserved neurogenesis under the stress of these anticancer drugs. In addition, PAN-811 showed neither inhibition of mitosis of the neural precursor cells by itself nor synergistic reduction of the proliferation of these cells with MTX/5-FU. Hence, PAN-811 may be a very promising candidate for the clinical control of MTX/5-FU-induced CICI and worthy of clinical investigation.
